# A new approach in insulin pump education improves glycemic outcomes: a randomized controlled trial

**DOI:** 10.1007/s00592-024-02340-y

**Published:** 2024-08-22

**Authors:** Karen Rytter, Anette Hougaard, Anne Grynnerup Skouboe, Nermin Serifovski, Ajenthen Gayathri Ranjan, Kirsten Nørgaard

**Affiliations:** 1 Copenhagen University Hospital - Steno Diabetes Center Copenhagen, Borgmester Ib Juuls Vej 83, Herlev, DK-2730 Denmark; 2 Department of Clinical Medicine, Faculty of Health and Medical Sciences, University of Copenhagen, Blegdamsvej 3B, Copenhagen N, DK-2200 Denmark; 3 Department of Clinical and Translational Research, Copenhagen University Hospital - Steno Diabetes Center Copenhagen, Borgmester Ib Juuls Vej 83, Herlev, DK-2730 Denmark

**Keywords:** Insulin pump, Diabetes self-management, Education, Empowerment, Time in ranges, Type 1 diabetes

## Abstract

**Aims:**

To address the scarcity of continued education for insulin pump users, we developed and evaluated a new program (NP) for individuals transitioning to a different insulin pump.

**Methods:**

In a randomized, controlled 3-month study, adults with type 1 diabetes and suboptimal HbA1c received either NP or usual care program (UC). The NP was designed in collaboration with representatives of the target group and incorporated technical training, case-based learning, and peer experience sharing – encompassing two group sessions, and two follow-up telephone calls. The UC included a single training session led by the pump company with hotline assistance (clinic) but no structured follow-up. The primary endpoint was the difference in time in range (TIR) (70–180 mg/dL (3.9–10.0 mmol/L)), measured by continuous glucose monitoring from baseline to 3 months post-course. Psychosocial self-efficacy was measured by the Diabetes Empowerment Scale (DES-SF).

**Results:**

Thirty-nine participants (median age 43, 74% female) were included. Mean TIR increased significantly in the NP group and remained unchanged in the UC group (between-group difference in change was 13.5% [95% CI: 4.0 to 22.9], *p* = 0.0064). Psychosocial self-efficacy improved and HbA1c decreased only significantly in the NP group.

**Conclusions:**

Applying a novel education program at pump transition significantly improved glycemic outcomes and self-efficacy.

**Supplementary Information:**

The online version contains supplementary material available at 10.1007/s00592-024-02340-y.

## Introduction

The field of diabetes technology, including insulin pumps, continuous glucose monitors (CGM) and their combination, is rapidly evolving, and use of insulin pumps is expanding in most countries. These technologies facilitate more accurate insulin dosing and frequent glucose monitoring, potentially easing life with diabetes and improving glycemic outcomes [1]. Guidelines and studies underscore the importance of structured support and training in new technologies, with increasing recognition that Diabetes Self-Management Education and Support (DSMES) should be delivered repeatedly during life and care transitions [2, 3]. Nevertheless, only a few papers have addressed the design, delivery, and effects of continuous insulin pump education [3, 4]. Insulin pump manufacturers offer valuable technical support hotlines and instruction programs for their products, but these services cannot replace the personal approach in DSMES including shared decision-making, self-care, and problem-solving in active collaboration with the healthcare team to improve clinical outcomes, health status, and quality of life [2, 3, 5, 6]. Getting used to new technology and ensuring its safe usage demands appropriate education programs, ideally also empowering insulin pump users to proactively upload their device data, systematically review it, and make relevant changes to pump settings. Recent studies have shown that pump users are interested in learning more about these aspects. Moreover, individuals who already review their data and adjust insulin pump settings themselves tend to have better HbA1c compared to those who rely solely on their healthcare providers for these adjustments [7, 8].

To facilitate the transition to a new insulin pump, we created an adaptive advanced education program for insulin pump users with diverse needs, aiming to enhance glycemic outcomes and self-efficacy. This first edition was targeted at transitioning to traditional insulin pumps, with the possibility of adjusting the program to other pump systems, such as automated insulin delivery systems (AID).

The objective of this study was to evaluate and compare the impacts of this novel continued education program, versus the usual technical training program, on glycemic outcomes and psychosocial self-efficacy in adults with Type 1 diabetes transitioning to a different insulin pump.

## Materials and methods

### Study design and participants

The study was conducted as a single-center randomized controlled study at Steno Diabetes Center Copenhagen, Denmark. Inclusion criteria were individuals aged 22 years or older, Type 1 diabetes for at least four years and insulin pump-treated for at least four years, HbA1c 7.5% (58 mmol/mol) or higher, and/or time in range (TIR) (70–180 mg/dL (3.9–10 mmol/L)) less than 55% measured for 30 days by their routinely used continuous or intermittently scanned glucose monitoring (CGM/isCGM) (if any). Participants were included if they had to replace a broken pump, the warranty had expired, and they wanted to switch from a different pump model to either a Tandem Basal IQ pump or Omnipod Dash. Regarding prior insulin pump education all participants had received basic education when starting insulin pump therapy and additional training around every 4–5 years at device change. Persons starting AID systems or wanting to use non-approved “Do it yourself” looping methods were excluded. Severe diabetes complications or comorbidities implying restrictions for HbA1c reduction were also exclusion criteria. Consecutively recruitment started on September 21, 2022, and the last participant’s last visit was 29, September 2023.

### Ethics and data protection

All participants provided informed consent. The study was exempt from review by the Capital Region of Denmark’s Research Ethics Committee (F-22,018,574). Data management was conducted following the General Data Protection Regulation and approved by the Danish Data Protection Agency (P-2022-221). All information on study participants was protected according to the laws on the processing of personal data and health. The electronic study database in REDCap was password-protected and located on the hospital network server.

### Intervention

The new education program (NP) was developed using a design thinking approach, involving several workshops and meetings with diabetes educators and the target group: adult experienced insulin pump users with Type 1 diabetes.

The NP included 5 parts (Overview S1): three one-to-one meetings (in-person or phone call) and two group sessions (3 h each). Total time consumption was approximately 8 h. The number of participants was a minimum of four and a maximum of eight in the group sessions. The curriculum included technical instructions regarding the new device and carb-counting refresher with practical training. Furthermore, daily life situations e.g., management of glucose regulation during exercise and disease were discussed together with data-upload analysis, principles of treatment adjustment, and hands-on adjustment of pump settings. Two different one-page handouts with short explanations and practical tips were shared at the course: A sports/physical activity guide and a device-specific guide for adjusting the insulin pump’s basic settings for insulin delivery. A user-centred agenda and problem-based learning methods (casework) were used to promote peer-to-peer experience sharing and dialogues about new possibilities and any personal plans for change in diabetes- and insulin pump self-management. Teaching and facilitation in the group sessions were handled by a diabetes nurse and a dietitian specialized in insulin pumps, group teaching, and facilitation.

The usual care program (UC) consisted of a single 3-hour group session, which included an introduction to the new pump, technical instruction, and practical advice regarding the device. The teaching was led by a representative from the pump manufacturer, with support from a diabetes nurse for the practical aspects and for ensuring correct insulin pump settings. While no specific follow-up was planned, participants could call the regular 24/7 diabetes nurse hotline for advice.

### Assignment and start of interventions

Participants were randomly assigned 1:1 to either NP or UC. To provide all participants with the NP, the participants assigned to UC were offered the NP after the study ended. Randomization was performed in REDCap, a web-based data management system, using a random allocation table uploaded by a person not involved in the study. The maximum time from baseline to intervention start was 40 days.

### Measures

#### Demographics and clinical characteristics

The following baseline clinical characteristics were obtained from the electronical medical record (EMR), which are updated at least every year: occurrence of late diabetes complications, body mass index, diabetes duration, insulin pump duration, hypoglycemia awareness status. Additionally, the number of severe hypoglycemic episodes requiring third-party assistance or medical intervention, and events with ketoacidosis within the previous 5 years, were retrieved. From the participants, the following information was obtained: their mean total daily insulin dose, insulin pump type, use of CGM, isCGM or finger-prick glucose monitoring (BGM) and key demographics, including sex, age, highest education level, occupation, and whether living with a partner. Lastly, HbA1c was measured at baseline, unless the participant had a HbA1c value measured within the last three weeks before inclusion.

#### Psychosocial health

Participants completed electronic questionnaires at baseline and the study end. Well-being and diabetes distress were monitored at baseline by the WHO5 Well-being index [9] and The Problem Areas in Diabetes Scale (PAID-5) [10]. The PAID-5 scores can range from 0 to 20, with higher scores suggesting greater diabetes-related emotional distress; a total score of ≥ 8 indicates possible diabetes-related emotional distress warranting further assessment [10]. The WHO5 raw score ranges from 0 to 25. The raw score is multiplied by 4 to give the final score where 0 represents the worst possible. Psychosocial self-efficacy was a secondary outcome and changes from baseline to study end were measured by the Diabetes Empowerment Scale Short Form (DES-SF) [11] (consisting of eight questions related to psychosocial self-efficacy). Possible responses for each item range from 1 to 5. A total DES-SF score can be calculated by summing all item scores and dividing by 8 (the total number of items) [11, 12].

#### Glucose metrics and insulin data

Glucose metrics were assessed using blinded and non-blinded Dexcom G6 CGM (Dexcom, San Diego, California, USA) for 10 days at study start and end. Blinded Dexcom CGMs were provided to non-Dexcom users. Glucose data were downloaded via the communication platform Glooko (Palo Alto, CA, USA) for review and analysis. The median active CGM time was slightly lower than the recommended (NP baseline, 9.5; UC baseline, 9.6; NP study end, 9.8; UC study end, 9.6 days). Metrics defined in the study were: Time in target range (TIR): 70–180 mg/dL (3.9–10 mmol/L); Time below range (TBR): <70 mg/dL (TBR1), < 54 mg/dL (TBR2) (< 3.9 mmol/L, < 3.0 mmol/L); Time above range (TAR): >180 mg/dL (TAR1), > 250 mg/dL (TAR2) (> 10 mmol/L, > 13.9 mmol/L); Time in tight range (TITR): 70–140 mg/dL (3.9–7.8 mmol/L); Glycemia Risk Index (GRI) calculated as (3.0 × %TBR2) + (2.4 × %TBR1) + (1.6 × %TAR2) + (0.8 × %TAR1) [13].

Hemoglobin A1c (HbA1c) was measured at baseline and study end.

Total daily insulin dose (TDD) was averaged at baseline and study end based on 14-day downloads from the participant’s insulin pump via Stenopool, a local communication platform at Steno Diabetes Center Copenhagen.

### Primary and secondary outcomes

The primary outcome was the difference between the groups (NP versus UC) in the change in TIR from baseline to study end.

Secondary outcomes were the difference in change for TAR, TITR, TBR, mean sensor glucose, glycemic variability, GRI, TDD, HbA1c, and difference in change in psychosocial self-efficacy (DES-SF) from baseline to study end.

### Statistics

No previous data were available to perform a formal power calculation. We estimated that 40 participants should be included for assessing primary outcome. Baseline characteristics were summarized for each group and presented as frequency with percentages for categorical outcomes and median with ranges for continuous outcomes. All outcomes were analyzed according to the intention-to-treat principles. In the presence of missing data, the last available observation was carried forward. A supplementary per protocol analysis was also done (Table S2, Table S4, Fig. S1, Fig. S2). Continuous outcomes following normal distribution were compared between intervention arms using the two-sided Student’s t-tests. Distribution of normality was assessed by the Shapiro Wilk test. If data were skewed, despite logarithmic transformation, a non-parametric Wilcoxon Signed Rank test was used. Categorical data were compared using a χ² test. A p-value of < 0.05 was considered statistically significant. Statistical analyses were conducted using R version 3.6.1.

## Results

### Baseline characteristics

**Fig. 1:**
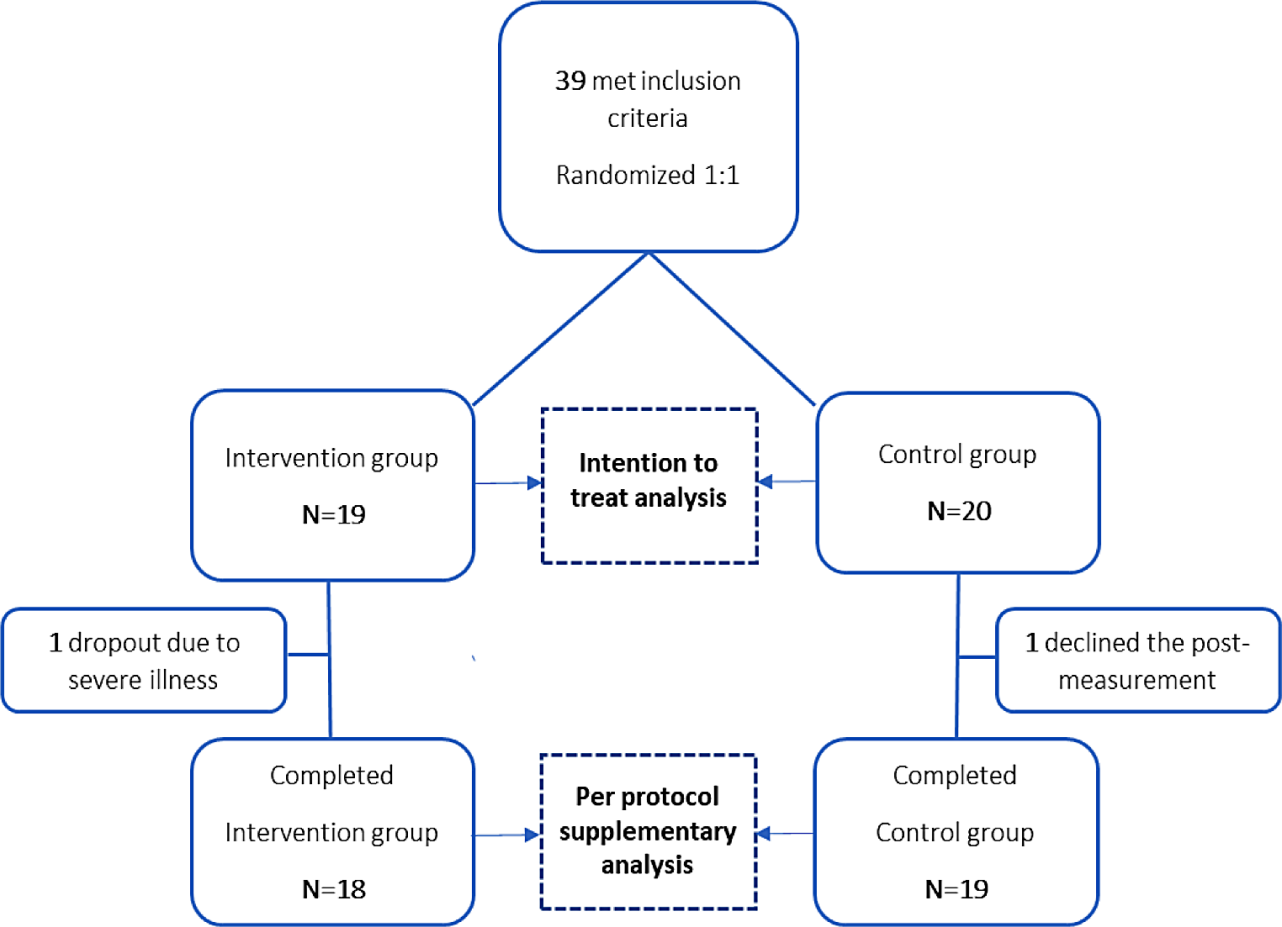
Flowchart for participants in the study

A total of 39 insulin pump users were included in the study, 19 in the NP group and 20 in the UC group. Two participants (one from each group) left the study before completion (Fig. 1). The two groups had similar baseline characteristics (Table 1). The participants represented a broad age span (20–40 years, 46%; 40–60 years, 36%; 60 + years; 18%) and most were female (74%). Median diabetes duration was 22 years and duration of insulin pump treatment was 11 years. Approximately half of the participants (51%) had microvascular complications, the majority (69%) had normal hypoglycemia awareness, median BMI was 26 kg/m^2^ and median HbA1c was 8.1% (65 mmol/mol). Real-time CGM was the most frequently used system for glucose monitoring, followed by isCGM. Four participants used blood glucose meter rather than CGM. After participants chose a new insulin pump, the distribution of devices was as follows: a tube pump was used by 11 from the UC group and 10 from the NP group; a patch pump was used by 9 from the UC group and 9 from the NP group (Table S1). Considering general psychosocial health, the median WHO-5 well-being index score was 52 and 49% had a score ≤ 50, which can indicate reduced well-being and risk of depression. With respect to diabetes related distress (PAID5), the median score was 7 and 49% had a score ≥ 8, indicating possible emotional diabetes distress.

**Table 1:** Demographic and clinical characteristics

Variables	All participants (*n* = 39)	NP group (*n* = 19)	UC group (*n* = 20)
Age, years	43 (22–71)	40 (23–70)	43 (22–71)
Sex, female	29 (74)	13 (68)	16 (80)
Male	10 (26)	6 (32)	4 (20)
Living with a partner	25 (64)	15 (79)	10 (50)
**Education**			
Primary school	1 (3)	1 (5)	0 (0)
High or vocational school	8 (21)	3 (16)	5 (25)
Short or medium higher	22 (56)	12 (63)	10 (50)
Long higher education	8 (21)	3 (16)	5 (25)
**Clinical characteristics**			
Diabetes duration, years	22 (6–44)	21 (7–44)	22 (6–43)
Pump use duration, years	11 (4–21)	12 (6–21)	9 (4–18)
Diabetic ketoacidosis, events in 5 years	2 (5)	0 (0)	2 (10)
Severe hypoglycemia, events in 5 years	1 (3)	0 (0)	1 (5)
Normal awareness	27 (69)	14 (74)	13 (65)
Impaired awareness	8 (21)	4 (21)	4 (20)
Unawareness	4 (10)	1 (5)	3 (15)
Microvascular complications	20 (51)	10 (53)	10(50)
Macrovascular complications	2 (5)	1 (5)	1 (5)
BMI	26 (20–47)	26 (20–39)	26 (20–47)
HbA1c, %	8.1 (7.3–10.6)	8.1 (7.3–10.3)	7.9 (7.5–10.6)
HbA1c, mmol/mol	65 (56–92)	65 (56–89)	63 (58–92)
**Psychosocial health**			
WHO5 score	52 (16–88)	56 (20–88)	50 (16–80)
Persons with WHO5 ≤ 50	19 (49)	9 (47)	10 (50)
PAID5 score	7 (2–20)	6 (2–15)	8 (2–20)
Persons with PAID5 ≥ 8	19 (49)	8 (42)	11 (55)
DES-SF score	3.6 (2–4)	3.2 (2–4)	3.6 (3–4)

Data are presented as frequency with percentages for categorical outcomes and median with ranges for continuous outcomes. *NP*, New Program; *UC*, usual care

### Glycemic outcomes

**Fig. 2:**
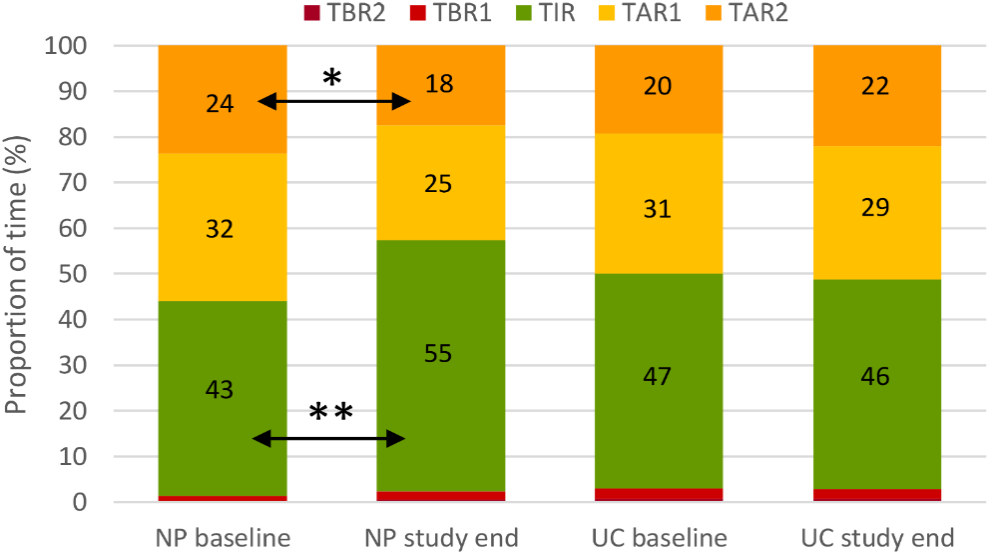
Mean sensor glucose time in ranges. Intention to treat analysis (*n* = 39), within-group changes from baseline to study end in Time in target range (TIR): 70–180 mg/dL (3.9–10 mmol/L); Time below range (TBR): <70 mg/dL, < 54 mg/dL (< 3.9 mmol/L, < 3.0 mmol/L); Time above range (TAR): >180 mg/dL, > 250 mg/dL (> 10 mmol/L, > 13.9 mmol/L). Data are shown for 39 participants. **Statistically significant with-in group change (*P* < 0.01); * Statistically significant with-in group change (*P* < 0.05)

From baseline to study end, mean TIR increased significantly by 12.3% (95% CI: 6.0 to 18.5) in the NP group and decreased by 1.2% (95% CI: -8.7 to 6.2) in the UC group (Fig. 2). The between-group difference in change was hence 13.5% (95% CI: 4.0 to 22.9, *p* = 0.0064). In Table 2, the glycemic outcomes are reported as medians and interquartile ranges. Figure 2 shows the mean values of the different time-in-ranges for each group before and after intervention.

**Table 2:** Primary and secondary glycemic outcomes

	NP group (*n* = 19)		UC group (*n* = 20)		*P*-value NP versus UC
Variables	Baseline	Study end	Baseline	Study end	Difference in change
**TAR:** >180 mg/dL (> 10 mmol/L) %	54.3 (46.1–63.6)	36.3 (33.6–50.2)	50.9 (41.2–59.5)	47.7 (42.5–66.2)	*P* = **0.0046**
**TAR2:** >250 mg/dL (> 13.9 mmol/L) %	22.6 (12.7–28.3)	12.2 (7.9–23.4)	17 (13.0–21.8)	19.7 (14–29.5)	*P* = **0.022**
**TAR1:** >180 mg/dL to 250 mg/dL (> 10 mmol/L to 13.9 mmol/L) %	31.4 (27.8–36.7)	26.7 (22.3–29.4)	33.2 (26.5–37)	30.2 (24.9–34.4)	*P* = 0.067
**TIR:** 70–180 mg/dL (3.9–10 mmol/L)%	45.7 (35.6–51.9)	58.9 (46.8–63.7)	47.5 (39.5–55.8)	48.3 (33.8–54)	*P* = **0.0064**
**TBR:** <70 mg/dL (< 3.9 mmol/L) %	0.3 (0.1–1.8)	1.2 (0.6–2.6)	1.3 (0.6–2.6)	1.2 (0.1–4.1)	*P* = 0.068^W^
**TBR1:** 54 mg/dL to < 70 mg/dL (3.0 mmol/L to < 3.9 mmol/L) %	0.4 (0.2–1.9)	1.3 (0.5–2.2)	1.4 (0.6–2.7)	1.3 (0.2–2.9)	*P* = 0.14^W^
**TBR2:** <54 mg/dL (< 3.0 mmol/L) %	0.0 (0.0–0.3)	0.1 (0.0–0.4)	0.2 (0.0–0.7)	0.1 (0.0–0.8)	*P* = 0.62^W^
**TITR: 70–140 mg/dL** (3.9–7.8 mmol/L)	23.4 (13.3–32.3)	32.2 (23.6–37.9)	27.7 (19.1–33.1)	25.9 (13.9–32.9)	*P* = **0.021**
**Mean sensor glucose** (mg/dL)	193 (178–214)	168 (160–195)	184 (177–204)	187 (175–213)	*P* = **0.0082**
**Mean sensor glucose** (mmol/mol)	10.7 (9.9–11.9)	9.3 (8.9–10.8)	10.2 (9.8–11.3)	10.4 (9.7–11.8)	*P* = **0.0082**
**SD sensor glucose** (mg/dL)	69 (58–83)	70 (56–76)	67 (61–72)	67 (63–76)	*P* = 0.20
**SD sensor glucose** (mmol/mol)	3.8 (3.2–4.6)	3.9 (3.1–4.2)	3.7 (3.4–4.0)	3.7 (3.5–4.2)	*P* = 0.20
**Coefficient of variation** (%)	35.5 (29.9–38.2)	36.4 (33.4–42.6)	34.2 (31.6–38.4)	36.7 (31.4–40.2)	*P* = 0.45
**Glycemia Risk Index**	62.7 (53.9–73.6)	46.3 (39.0–76.5)	58.9 (54.1–68.5)	66.6 (55.1–75.6)	*P* = **0.031**
**HbA1c** (IFCC) (mmol/mol)	65 (63–68)	58 (54–65)	64 (61–70)	64 (59–72)	*P* = **0.019**^L^
**HbA1c** (NGSP) (%)	8.1 (7.9–8.4)	7.5 (7.0–8.1)	8.0 (7.7–8.6)	8.0 (7.5–8.8)	*P* = **0.019**^L^
**Total daily insulin dose** (U/day)	39.9 (37.1–56.5)	40.9 (36.5–51.2)	40.8 (34.8–52.6)	43.6 (31.2–53.1)	*P* = 0.87^W^

Data are shown with median and interquartile ranges (IQR). Distribution of glucose values (% time in ranges, 10 days CGM): *TAR* Time above range; *TIR* Time in target range; *TBR* Time below range, *TITR* Time in tight range; Glycemia Risk Index is calculated as (3.0 × %TBR2) + (2.4 × %TBR1) + (1.6 × %TAR2) + (0.8 × %TAR1). Total daily insulin is insulin pump upload data from a period of 14 days. Last column Student’s t-test results; ^W^ Wilcoxon Signed Rank test was used instead of Student’s t-test. ^L^ data were log-transformed

**Fig. 3:**
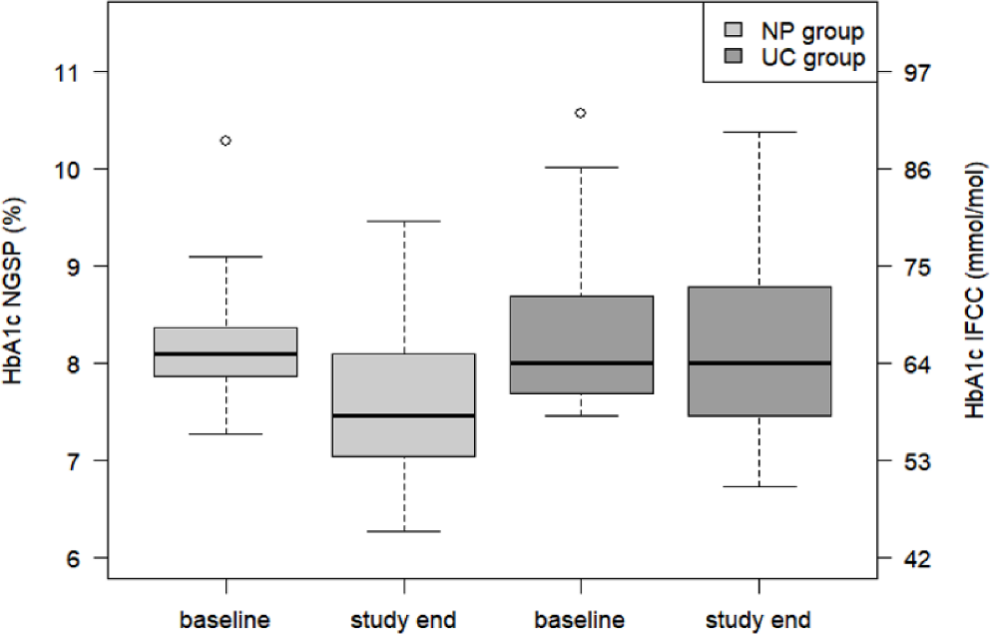
Distribution of HbA1c levels at baseline and study end. Intention to treat analysis (*n* = 39). *NP* New program (*n* = 18); *UC* usual care (*n* = 19)

Regarding the secondary outcomes, mean TAR decreased in the NP group by 13.3% (95% CI: -19.7 to -7.0) and remained unchanged in the UC group (between-group difference, *p* = 0.0046) (Table 2). TBR did not change significantly in any of the groups from baseline to study end, but a trend of increased TBR in the NP group was observed (Table 2; Fig. 2). TITR increased significantly in the NP group (9.9%, 95%CI: 1.6 to 18.4) and not in the UC group (between-group difference, *p* = 0.021). Average sensor glucose decreased significantly in the NP group and not in the UC group (between-group difference, *p* = 0.0082). The coefficient of variation and SD did not change significantly in any of the groups nor did TDD (Table 2). HbA1c decreased significantly in the NP group and not in the UC group (between-group ratio = 0.89, *P* = 0.019) (Table 2; Fig. 3).

### Changes in psychosocial self-efficacy

Psychosocial self-efficacy score measured by DES increased significantly in the NP group from baseline to study end from 3.4 to 3.7 points (*P* < 0.01). For the UC group, the score remained unchanged. DES score increased in 89% of participants in the NP group and 42% in the UC group (*P* < 0.01), while scores for the remaining participants decreased or were unchanged (Table S3).

## Discussion

In this randomized study, we assessed the effectiveness of our 8-hour newly developed continuing education program, specifically designed for insulin pump users transitioning to a different non-automated insulin pump. Participants in the new program demonstrated significant glycemic improvements compared to those in the shorter usual program. Additionally, only the NP group showed significant enhancement in psychosocial self-efficacy. These findings underscore the importance of diabetes education, aligning with recent guidelines [2, 14]. The guidelines highlight the importance of accessible diabetes technology in improving the lives and health of persons with diabetes and stress the ongoing need for education and support in diabetes self-management during the transition to new technologies. Limited randomized controlled trials describe and thoroughly evaluate educational interventions in diabetes technology. Particularly scarce are publications evaluating the effects of insulin pump self-management programs provided beyond the initial training at insulin pump therapy start-ups. However, the German INPUT-study [15] – an RCT with 268 participants, published in 2018, evaluated the effects of a re-education program with a broad curriculum. This program addressed not only insulin pump self-management but also emotional and motivational aspects of life with diabetes. The study demonstrated a between-group difference of 0.22% in HbA1c, favoring the intervention group after 6 months, along with improvements in psychosocial outcomes such as diabetes distress. The INPUT program ran for twelve weeks, with 90-minute sessions once or twice a week. Not every healthcare system will have the capacity to allocate the resources for such a program. Furthermore, the question is whether those in most need of diabetes education have the resources to devote the necessary time to such an extensive intervention. A recent study on CGM education had a design more comparable to ours, closely aligned in terms of time frame and endpoints. This study evaluated an education and support intervention including three one-to-one sessions (duration of 30 to 120 min) [16]. The outcomes of this study mirrored our results, demonstrating comparable improvements in TIR and HbA1c. The authors attributed the positive results to the personalized nature of one-to-one training [16]. While we acknowledge the importance of DSMES taking an individual starting point, we also recognize the potential benefits of group-based education. The exchange of experiences with peers can serve as a powerful tool for gaining new insights, emotional support, and motivation [17]. These mechanisms are integral to our approach, and the improvement in psychosocial self-efficacy in the NP group in our study might be linked. At study onset, half of all the participants had WHO5 and PAID-5 scores indicative of low general well-being and possible diabetes distress. Interacting with peers might reduce the burden, as supported by studies linking social support interventions with reduced diabetes distress, and better self-care in diabetes [17, 18]. Unfortunately, we did not specifically measure diabetes distress post-intervention, as our program did not target it explicitly, and the study duration was considered insufficient to observe a significant difference.

The strengths of our study are the close relationship to the clinical care setting, the experienced diabetes educators involved in designing and conducting the program, and the collaboration with the target group in developing the intervention, making it feasible and relatively easy to implement in comparable healthcare settings.

The idea of our study originated in clinical practice. Therefore, the design and execution were planned within this setting, respecting the constraints of available resources and policies within the framework of a public healthcare system. These conditions played a crucial role in determining the study’s timeline and the number of participants eligible for inclusion. For instance, at the study’s inception, the prevailing practice dictated that not all users could be allocated CGM. Those receiving a Tandem insulin pump would initially start with Basal IQ, with the possibility of later upgrading to Control-IQ. After study start, advancements in diabetes technology and new policies have enabled broader access, allowing all persons with type 1 diabetes to be equipped with a CGM and more to proceed to AID treatment. Consequently, extending the follow-up duration in the study became unfeasible, posing a clear limitation as it remains uncertain whether the positive results are enduring. Secondly, the limited sample size prevented subgroup analyses on predictors for a positive outcome of the education program or evaluating needs for changes in the program for sub-groups. Lastly, it should be considered if this approach to insulin pump self-management education is feasible and effective in other settings. Testing it in other centers or through a multicenter study would be relevant.

The future is undoubtedly moving towards AID treatment, but, we expect our program to remain relevant. Considering economic factors, many insulin pump users will continue to use traditional pumps for the foreseeable future. Importantly, our education program’s structure is well-suited for integration with new technologies such as AID pump systems, making it valuable with minor adjustments in facilitating the transition to these systems.

In conclusion, we created and evaluated a new continuing education program, designed for individuals transitioning to a different type of traditional insulin pump. The program was found to positively impact both glycemic outcomes and self-efficacy. The study sample size was relatively small, and the follow-up period was limited to three months. More extensive and longer-term studies would be relevant to detect long-term effects, uncover underlying mechanisms for effects and evaluate customized versions incorporating new technologies. Nevertheless, the current study demonstrated a significant impact of a relatively short and feasible insulin pump education program. This underscores the role of DSMES in diabetes technology and addresses the gap in research within this field.

## Electronic supplementary material

Below is the link to the electronic supplementary material.

Supplementary Material 1

Supplementary Material 2
