# Supplementary material for: A new approach in insulin pump education improves glycemic outcomes: a randomized controlled trial
Source: Acta Diabetol. 2024 Aug 22;62(1):87–94. doi: 10.1007/s00592-024-02340-y (PMC11772455; doi:10.1007/s00592-024-02340-y)
Supplement: Supplementary file 2 — Supplementary Material 2 [file 592_2024_2340_MOESM2_ESM.pdf]

Supplementary information

A new approach in insulin pump education improves glycemic outcomes: a randomized controlled trial; Acta Diabetologica

Karen Rytter[a,b], Anette Hougaard[a], Anne Grynnerup Skouboe[a], Nermin Serifovski[a], Ajenthen Gayathri Ranjan[a], Kirsten Nørgaard[a,b]

Corresponding Author: Karen Rytter, karen.rytter@regionh.dk

Copenhagen University Hospital - Steno Diabetes Center Copenhagen, Borgmester Ib Juuls Vej 83, DK-2730 Herlev, Denmark

Author affiliations:
[a] Copenhagen University Hospital - Steno Diabetes Center Copenhagen, Borgmester Ib Juuls Vej 83, DK-2730 Herlev, Denmark.
[b] Department of Clinical Medicine, Faculty of Health and Medical Sciences, University of Copenhagen, Blegdamsvej 3B, DK-2200 Copenhagen N, Denmark.

Overview Supplementary material

**Overview S1**	Education program	3
**Table S1**	Technology characteristics	4
**Table S2**	Primary and secondary glycemic outcome – Per-protocol analysis	5
**Table S3**	Psycho-social self-efficacy difference from baseline to study end – Intention to treat analysis (n=39)	6
**Table S4**	Psycho-social self-efficacy difference from baseline to study end – Per-protocol analysis (n=37)	6
**Fig. S1**	Mean glucose time in ranges, within-group changes – Per-protocol analysis (n=37)	7
**Fig. S2**	Distribution of HbA1c levels baseline and study end – Per-protocol analysis (n= 37)	7
**Fig. S3**	Flow for data collection and participation in the NP and the UC program	8

Overview S1

New education program (aligned in user workshops)				
**Consultation** (1 hour)<br>Approx. 1 month before the pump course	**Group course (GC) part 1**<br>3 hours	**Follow-up telephone call** 1 week after GC part 1	**Group course part 2**<br>3 hours 2 weeks after GC part 1	**Follow-up consultation**<br>4 weeks after GC part 2
Choice of new insulin pump, individual visit with the nurse<br><br>**Content:**<br>• Insulin pump selection<br>• Eventual problems with the actual device (e.g., operating the pump, infusions set, skin issues)<br>• clarification of expectations and needs (self-care, insulin pump management skills, psycho-social needs)<br>• plan for the course<br>• Update insulin pump settings	**Teaching and facilitation:** Diabetes nurse specialist and dietitian with help from a product specialist from the manufacturer.<br><br>**Contents:**<br>• Introduction to the new insulin pump<br>• Practice set change & basic pump functions<br>• Brush-up carb counting<br>• Presentation - what do we know about insulin pump use and the association with blood glucose<br>• Which topics are relevant in the group for the next time? "Participant-selected homework"<br>• Questions	Individual telephone follow-up 15-20 minutes with diabetes nurse. Attendance in outpatient clinic if needed<br><br>**Content:**<br>• Feeling safe and confident with the new device?<br>• Technical questions about the pump<br>• The user uploads the pump at home before the consultation<br>• Pump settings are revised and adjusted if necessary	**Teaching and facilitation:** Diabetes nurse/dietitian<br><br>**Contents:**<br>• Principles for adjusting the pump settings (Casework)<br>• The group's topics, for example:<br>• Management of physical activity/sport (Casework)<br>• Use of combined/extended bolus<br>• Prevent device-related skin problems<br>• Managing low blood glucose<br>• Kinked sets, sickness, and ketones<br>Evaluation of group course part one and part two	Individual telephone follow-up 30 minutes with diabetes nurse, attendance in outpatient clinic if needed.<br><br>**Content:**<br>• Revision, and adjustment of the insulin settings<br>• follow-up on own focus areas, e.g., handling glucose/ exercise, hypo-management<br><br>*Only if relevant for the insulin pump user, but the consultation is offered to everyone*

Table S1 – Technology characteristics

	Before pump change<br>All (n=39)	New pump<br>All (n=39)	New pump<br>NP group<br>(n=19)	New pump<br>UC group<br>(n=20)
**Patch pump**	19	18	9	9
**Tube pump**	20	21	10	11
**CGM**	22	24	12	12
**Flash libre**	5	5	2	3
**Libre 2**	8	6	3	3
**BG meter**	4	4	2	2

New program (NP), Usual care program (UC)

In the category "New pump", patch pumps refer to Omnipod Dash and tube pumps refer to Tandem Basal IQ.

Insulin pump types used before pump change included the patch pumps Medtrum, Omnipod Eros, Omnipod Dash and the tube pumps Medtronic 640G, Medtronic VEO, and Ypsopump.

Additional info. Six participants used sensor- augmented pumps (640G) at baseline, they chose a different sensor-augmented pump (Tandem Basal IQ). After randomization, these participants were distributed with 2 participants in the UC group and 4 participants in the NP group.

Table S2 – Primary and secondary glycemic outcome – per-protocol analysis

	NP group (n=18)		UC group (n=19)		P-value NP versus UC
Variables	**Baseline**	**Study end**	**Baseline**	**Study end**	**Difference in change**
**TAR: >180 mg/dL (>10 mmol/L) %**	51.3 (46 - 61.8)	36.3 (33.3 - 43.9)	50.4 (40.6 - 57.6)	45.4 (42.3 - 64)	**P=0.0044**
**TAR2: >250 mg/dL (>13.9 mmol/L) %**	21.5 (12.6 - 27.3)	12.1 (7.0 - 14.8)	16.6 (12.7 - 19.7)	19.5 (13.1 - 28.5)	**P=0.022**
**TAR1:** >180 mg/dL to 250 mg/dL (>10 mmol/L to 13.9 mmol/L) %	31.7 (27.5 - 36.8)	25.5 (22.3 - 28.5)	32.7 (26.4 - 36.9)	30.1 (24.8 - 33.9)	P=0.064
**TIR:** 70-180 mg/dL (3.9–10 mmol/L) %	46.9 (38.2 - 52)	59.1 (52.3 - 64.1)	47.6 (41.1 - 56.3)	49 (36 - 54.4)	**P=0.0061**
**TBR: <70 mg/dL (<3.9 mmol/L) %**	0.5 (0.2 – 2.0)	1.3 (0.7 - 2.7)	1.3 (0.7 - 3.1)	1.7 (0.1 - 4.2)	P=0.068^{W}
**TBR1:** <54mg/dL to <70 mg/dL (3.0 mmol/L to <3.9 mmol/L) %	0.6 (0.3 - 2.1)	1.4 (0.7 - 2.3)	1.4 (0.8 - 3.1)	1.9 (0.2 - 3)	P=0.15^{W}
**TBR2: <54mg/dL (<3.0 mmol/L) %**	0.0 (0.0 - 0.4)	0.1 (0.0 - 0.4)	0.2 (0.1 - 0.7)	0.1 (0.0 - 0.8)	P= 0.58^{W}
**TITR: 70-140 mg/dL (3.9-7.8 mmol/L)**	23.7 (14.6 - 32.3)	32.5 (26.2 - 38.8)	27.8 (19.6 - 33.3)	26.4 (14.8 - 33)	**P=0.021**
**Mean sensor glucose** (mg/dL)	191(178-211)	167 (160-180)	184 (176 - 198)	187 (173 – 207)	**P=0.0079**
**Mean sensor glucose** (mmol/mol)	10.6 (9.9 - 11.7)	9.3 (8.9 – 10.0)	10.2 (9.8 – 11.0)	10.4 (9.6 - 11.5)	**P=0.0079**
**SD sensor glucose** (mg/dL)	67 (58 – 83)	67 (56 – 76)	65 (59 -74)	67 (63 -76)	P=0.20
**SD sensor glucose** (mmol/mol)	3.7 (3.2 - 4.6)	3.7 (3.1 - 4.2)	3.6 (3.3 - 4.1)	3.7 (3.5 - 4.2)	P=0.20
**Coefficient of variation** (%)	35.7 (31.2 - 38.7)	36.8 (33.7 - 42.6)	35.3 (31.5 - 38.4)	37.1 (31.3 - 40.3)	P=0.45
**Glycemia Risk Index**	62.2 (53.8 - 72.1)	45.8 (37.5 - 74.4)	56.9 (53.5 - 67)	66.2 (54.2 - 73.5)	**P=0.030**
**HbA1c** (IFCC) (mmol/mol)	65 (62.2 - 68)	57 (53.2 - 62.5)	63 (60.5 - 71.5)	63 (58 - 72.5)	**P=0.019^{L}**
**HbA1c** (NGSP) (%)	8.1 (7.8 - 8.4)	7.4 (7 - 7.9)	7.9 (7.7 - 8.7)	7.9 (7.5 - 8.8)	**P=0.019^{L}**
**Total daily insulin dose** (U/day)	39.9 (37.1 - 56.5)	40.9 (36.5 - 51.2)	40.8 (34.8 - 52.6)	43.6 (31.2 - 53.1)	P=0.87^{W}

Data are shown with median and interquartile ranges (IQR). Distribution of glucose values (% time in ranges, 10 days CGM): TAR, Time above range; TIR, Time in target range; TBR, Time below range, TITR, Time in tight range; Glycemia Risk Index is calculated as (3.0 × %TBR2) + (2.4 × %TBR1) + (1.6 × %TAR2) + (0.8 × %TAR1). Total daily insulin is insulin pump upload data from a period of 14 days. Last column Student's t-test results; ^{W} Wilcoxon Signed Rank test was used instead of Student's t-test. ^{L} data were log-transformed.

Table S3 – Psychosocial self-efficacy difference from baseline to study end – Intention to treat analysis (n=39)

	Increase in score n (%)	Unchanged or decrease in score, n (%)
NP group (n=19)	16 (84%)	3 (16%)
UC group (n=20)	8 (40%)	12 (60%)

Measured by Diabetes Empowerment Scale short form

Pearson's Chi-squared test with Yates' continuity correction
X-squared = 6.2868, df = 1, p-value = 0.01216

Table S4 – Psychosocial self-efficacy difference from baseline to study end – Per protocol analysis (n=37)

	Increase in score n (%)	Unchanged or decrease in score, n (%)
NP group (n=18)	16 (89%)	2 (11%)
UC group (n=19)	8 (42%)	11 (58%)

Measured by Diabetes Empowerment Scale short form

Pearson's Chi-squared test with Yates' continuity correction
X-squared = 6.9428, df = 1, p-value = 0.008416

Fig. S1 - Mean glucose time in ranges, within-group changes – Per-protocol analysis (n=37)

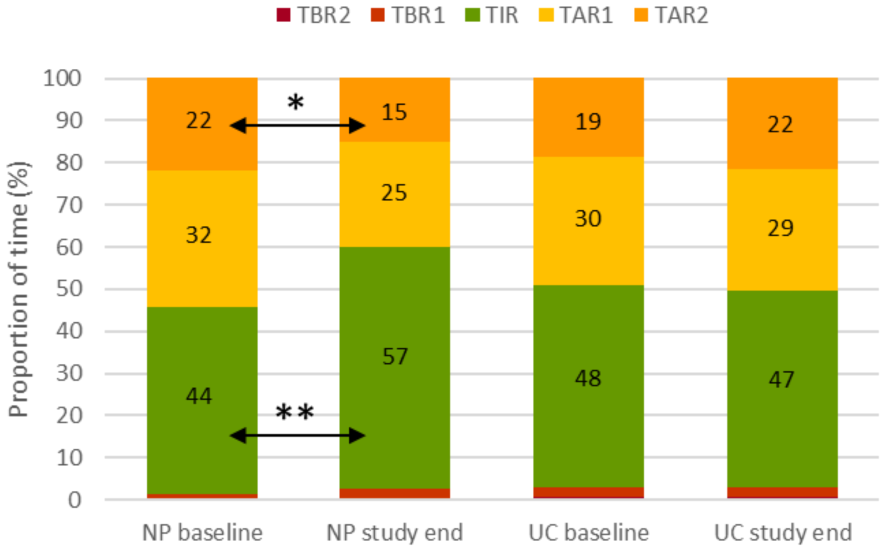

Fig. S1 Mean sensor glucose time in ranges. Per-protocol analysis (n=37), within-group changes from baseline to study end in Time in target range (TIR): 70-180 mg/dL (3.9–10 mmol/L); Time below range (TBR): <70 mg/dL, <54mg/dL (<3.9 mmol/L, <3.0 mmol/L); Time above range (TAR): >180 mg/dL, >250 mg/dL (>10 mmol/L, >13.9 mmol/L). Data are shown for 39 participants. ** Statistically significant with-in group change (P< 0.01); * Statistically significant with-in group change (P< 0.05).

Fig. S2 - Distribution of HbA1c levels baseline and study end – Per-protocol analysis (n= 37)

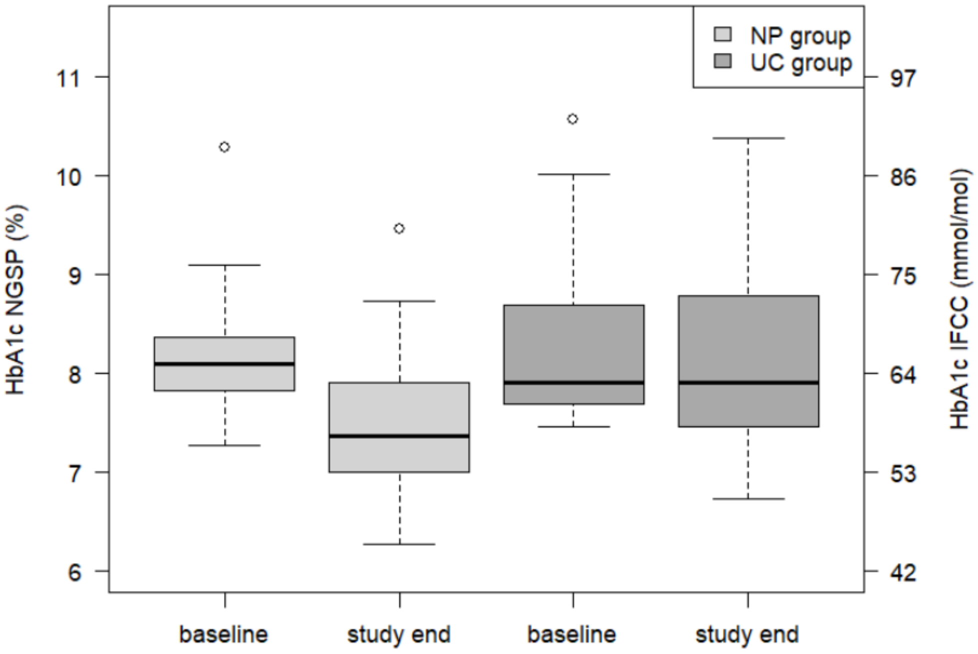

Fig. S2 Distribution of HbA1c levels at baseline and study end. Per-protocol analysis (n= 37). NP, New program (n=18); UC, usual care (N=19).

Fig. S3 Flow for data collection and participation in the NP and the UC program

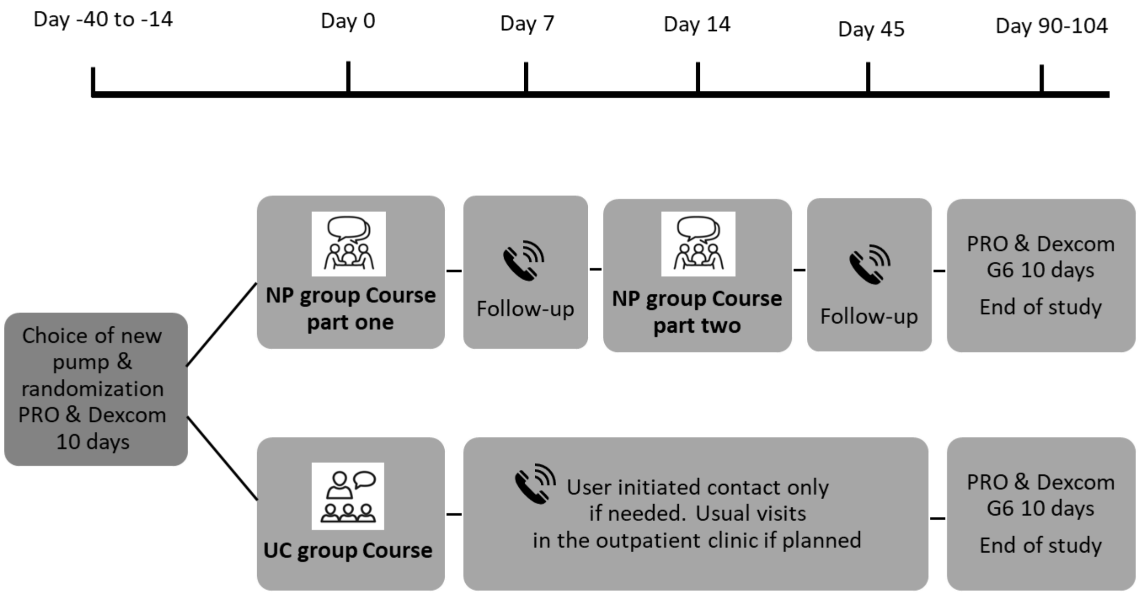

PRO, person reported outcome

Note, the UC group were offered additional education similar to the NP group after study end.
